# Synthesis of a Potent Aminopyridine-Based nNOS-Inhibitor by Two Recent No-Carrier-Added ^18^F-Labelling Methods

**DOI:** 10.3390/molecules21091160

**Published:** 2016-09-01

**Authors:** Christian Drerup, Johannes Ermert, Heinz H. Coenen

**Affiliations:** Institut für Neurowissenschaften und Medizin, INM-5: Nuklearchemie, Forschungszentrum Jülich, 52425 Jülich, Germany; ch.drerup@fz-juelich.de (C.D.); h.h.coenen@fz-juelich.de (H.H.C.)

**Keywords:** radiofluorination, radiopharmaceuticals, nNOS-inhibitor, iodonium ylides, copper-mediated labelling

## Abstract

Nitric oxide (NO), an important multifunctional signaling molecule, is produced by three isoforms of NO-synthase (NOS) and has been associated with neurodegenerative disorders. Selective inhibitors of the subtypes iNOS (inducible) or nNOS (neuronal) are of great interest for decoding neurodestructive key factors, and ^18^F-labelled analogues would allow investigating the NOS-function by molecular imaging with positron emission tomography. Especially, the highly selective nNOS inhibitor 6-((3-((3-fluorophenethylamino)methyl)phenoxy)methyl)-4-methylpyridin-2-amine (**10**) lends itself as suitable compound to be ^18^F-labelled in no-carrier-added (n.c.a.) form. For preparation of the ^18^F-labelled nNOS-Inhibitor **[^18^F]10** a “build-up” radiosynthesis was developed based on a corresponding iodonium ylide as labelling precursor. The such activated phenethyl group of the compound was efficiently and regioselectively labelled with n.c.a. [^18^F]fluoride in 79% radiochemical yield (RCY). After conversion by reductive amination and microwave assisted displacement of the protecting groups, the desired nNOS-inhibitor was obtained in about 15% total RCY. Alternatively, for a simplified “late-stage” ^18^F-labelling procedure a corresponding boronic ester precursor was synthesized and successfully used in a newer, copper(II) mediated n.c.a. ^18^F-fluoro-deboroniation reaction, achieving the same total RCY. Thus, both methods proved comparatively suited to provide the highly selective NOS-inhibitor **[^18^F]10** as probe for preclinical in vivo studies.

## 1. Introduction

Nitric oxide (NO) is an important signalling molecule and a unique mediator of a variety of physiological and pathological processes in the human body [1]. NO is synthesized endogenously from l-arginine by the enzyme nitric oxide synthase (NOS) existing in three isoforms [2]. There are two low-output isoforms of the NOS family, producing NO constitutively: the neuronal (nNOS) and the endothelial (eNOS) isoform. The nNOS generates NO as a neurotransmitter in brain and peripheral nerve cells, whereas eNOS derived NO is involved in the regulation of blood pressure, primarily in vascular endothelial cells. In contrast, the third, inducible (iNOS) isoform is induced by various inflammatory stimuli (e.g., endotoxin) and plays a crucial role in the immune system’s defence against pathogens and tumour cells. Once expressed, the iNOS can generate high amounts of NO up to μM concentrations and for a prolonged period of time such as for hours or days [3].

Various studies have shown that dysregulation of iNOS derived NO is closely implicated in numerous acute and chronic inflammatory diseases, e.g., septic shock [4], asthma [5] and multiple sclerosis [6,7,8,9]. In addition, iNOS activity has also been found in many tumours [9,10,11]. Equally important, earlier studies have revealed that overproduction of NO by the nNOS is associated with neurodegenerative diseases, including Parkinson’s [12], Alzheimer’s [13] and Huntington’s diseases [14], multiple sclerosis [15], headaches [16] and neuronal damage in stroke [17]. Thus, assessing the in vivo activity of iNOS or nNOS would provide insight into a multitude of physiologic and pathologic processes. Therefore, isoform-selective NOS imaging using suitable positron emitting inhibitors and non-invasive positron emission tomography (PET) would particularly be desirable and probably be of tremendous value for the study and treatment of NO related diseases. Given the central role of nNOS in a variety of diseases, PET may provide new avenues for diagnostics, the clinical management of affected patients, and it may facilitate the drug discovery and development process for new therapies that target NOS activity.

**Figure 1:**
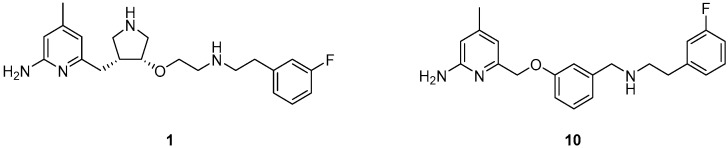
Novel NOS inhibitors: 6-(((3*R*,4*R*)-4-(2-((3-fluorophenethyl)amino)ethoxy)pyrrolidin-3-yl)methyl)-4-methylpyridin-2-amine (**1**) [23] and 6-((3-(((3-fluorophenethyl)amino)methyl)phenoxy)methyl)-4-methylpyridin-2-amine (**10**) [25].

Because of the central role of iNOS and nNOS in NO related diseases, numerous efforts have been made to develop effective inhibitors as pharmaceuticals. This ranges from nonselective l-arginine analogues [18] to selective inhibitors reported recently [19,20], that do especially not interfere with eNOS, to ensure proper functioning of the cardiovascular system [19,21]. Silverman and co-workers developed different aminopyridine-based molecules conceived as inhibitors for targeting the neuronal NOS [22,23,24]. In this context 6-(((3*R*,4*R*)-4-(2-((3-fluorophenethyl)amino)ethoxy)pyrrolidin-3-yl)methyl)-4-methylpyridin-2-amine (**1**, Figure 1) shows high potency with a *K_i_* value of 7 nM for nNOS and very high selectivity over iNOS (806-fold) and eNOS (2667-fold) [23].

In addition to a high potency and excellent isozyme-selectivity, the blood-brain-barrier permeability is a major challenge in the development of nNOS inhibitors. The same authors speculated that the therapeutic utility of **1** is limited by poor membrane permeability due to the two secondary amino groups [25]. Therefore, different strategies were applied to lower the possibility of forming positive charges at physiological pH at these positions and to improve the bioavailability [24]. Replacing the pyrrolidine group of **1** with a benzyl group lowered not only proton acceptor characteristics, but reduced also the number of chiral centres and enabled an easier synthesis. Especially the derivative 6-((3-((3-fluorophenethylamino)methyl)phenoxy)methyl)-4-methylpyridin-2-amine (**10**, Figure 1) combined comparable high potency and selectivity for nNOS (*K_i_* = 40 nM; 147-fold selectivity over iNOS and 261-fold over eNOS) [25] with a high probability to penetrate the blood-brain-barrier, thus satisfying the requirements for its application as a potential nNOS-radio inhibitor.

The aim of this study was to develop a synthesis for the preparation of the ^18^F-labelled inhibitor **[^18^F]10** as potential molecular imaging probe for the pathophysiologically interesting nNOS-isozyme and for further pharmacological and preclinical studies in order to localize, identify and quantify neurodestructive processes.

## 2. Results and Discussion

### 2.1. Synthesis of Reference Compounds and Precursors for a “Build-Up” Labelling Procedure

**Scheme 1:**
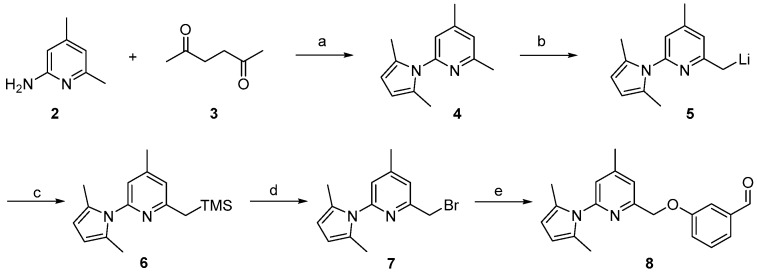
Synthesis of the aldehyde **8** as building block for the synthesis of the reference compound **10** and the precursor **19**. *Reagents and conditions*: (a) *p*-TsOH, toluene, reflux, 48 h; (b) *n*-BuLi, THF, 2 h, −78 °C to 0 °C; (c) TMSCl, THF, 1 h, 0 °C; (d) BrF_2_CCF_2_Br, CsF, DMF, 3 h, room temperature; (e) 3-hydroxybenzaldehyde, K_2_CO_3_, acetone, 4 h, 60 °C; steps a–d, see [26]; step e, see [25].

Previous work on the synthesis of potential inhibitors of neuronal nitric oxide synthase with rather simple structures provide a suitable preparative way for the reference compound **10** which is needed for an unambiguous identification of the desired radiolabelled nNOS-tracer. The following synthesis concept was used (see Scheme 1). After pyrrole-protection of 2-amino-4,6-dimethylpyridine **2** and deprotonation of **4**, the lithiated compound **5** was quenched with trimethylsilylchloride (TMSCl), according to an earlier report [26]. The resulting compound **6** was bromodesilylated, yielding **7** as starting material for coupling with 3-hydroxybenzaldehyde in compliance with the literature [25].

**Scheme 2:**
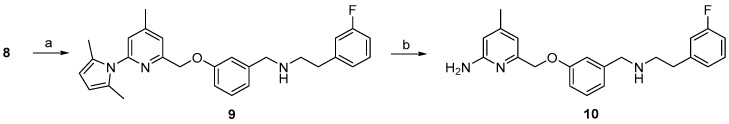
Preparation of the nNOS inhibitor **10** as reference compound. *Reagents and conditions*: (a) NaBH(OAc)_3_, 3-fluorophenethyl amine, MeOH, 3 h, room temperature; (b) NH_2_OH·HCl, EtOH/H_2_O (2:1), 20 h, 100 °C; step (b), see [25].

The pyrrole protected reference compound **9** was obtained after reductive amination of **8** with 3-fluorophenethylamine. Using methanol as protic solvent instead of the recommended 1,2-dichloroethane [25], increased the yield from 64% to 89%, as shown in Scheme 2, due to complete reduction of the imine intermediate. After deprotection of the amino group with NH_2_OH∙HCl, according to the literature [25], the nNOS inhibitor **10** was obtained and used as reference compound for further High performance liquid chromatography (HPLC) measurements.

Different approaches were considered for the radiosynthesis of the desired nNOS tracer **[^18^F]10**. The first concept was planned as a multistep radiosynthesis based on the use of an iodonium ylide as precursor for labelling. Former studies have shown that aryliodonium ylides are a promising alternative to the well-known diaryliodonium salts for the direct preparation of complex, electron rich no-carrier-added (n.c.a.) [^18^F]fluoroarenes [27,28]. However, recent findings about the reaction of ylides had to be considered. Especially, an unexpected formation of regioisomers as reported by Cardinale et al. [28] was a hint to additionally investigate the influence of the substitution pattern on the radiochemical yield during n.c.a. ^18^F-radiofluorination of iodonium ylide precursors. Therefore, the positional isomers **14o** and **14p** were synthesized in addition to the required iodonium ylide **14m** which was needed as precursor for the conversion to the desired nNOS inhibitor **10**.

**Scheme 3:**
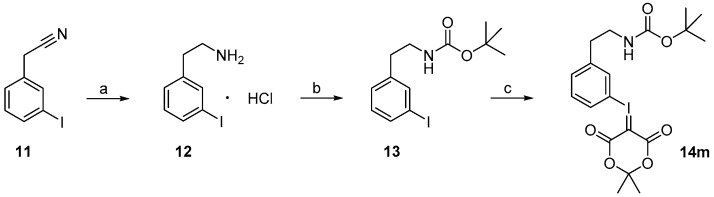
Synthesis of the iodonium ylide precursor **14m**. *Reagents and conditions*: (a) 1. BH_3_·THF, 70 °C, 2 h; 2. EtOH, HCl·Et_2_O, 0 °C, 5 min; (b) Boc_2_O, NaHCO_3_, MeOH, 50 °C, ultrasound, 30 min; (c) 1. *m*-CPBA, DCM, RT, 2 h; 2. KOH, Meldrum’s acid, room temperature, 15 min; 3. combined mixture of 1. and 2. (analogous to [29]).

Starting from 3-iodophenylacetonitrile **11** the corresponding primary amine **12** was obtained by reduction with borane. It was Boc-protected and subsequently converted with *m*-CPBA, potassium hydroxide and Meldrum’s acid to the corresponding iodonium ylide precursor **14m** in analogy to the literature [29] as shown in Scheme 3. The purification of the crude product was performed by column chromatography yielding 43% of the *meta*-substituted iodonium ylide precursor **14m**.

The corresponding fluorophenethylcarbamates **15o**, **15m** and **15p** (**13** with a fluorine in place of an iodine atom) were synthesized as reference compounds for radio-HPLC-identification, starting from commercially available fluorophenethylamines and were obtained in high yields of about 96%–98%.

### 2.2. ^18^F-Radiosynthesis of the nNOS-Inhibitor ***10*** via the “Build-Up” Procedure

As mentioned, Cardinale et al. observed the formation of two regioisomers during the preparation of the receptor ligand 4-((4-[^18^F]fluorophenoxy)phenylmethyl)piperidine with the corresponding *para*-substituted iodonium ylide precursor [28]. For a “build-up” synthesis of desired nNOS-tracer, however, the radiofluorinated intermediate **[^18^F]15m** must be synthesised in high radiochemical yield and high purity, avoiding any radioactive side product. Thus, for a careful examination of the n.c.a. radiofluorination of the iodonium ylide precursor **14m** as starting material for “build-up” of **[^18^F]9**, the *ortho*- and *para*-substituted ylides **14o** and **14p** were also labelled with n.c.a. [^18^F]fluoride at 110 °C in DMF with Kryptofix^®^2.2.2 (K_222_) and potassium carbonate ([^18^F]KF/K_222_), according to general procedures [28].

Comparing the generated products, the *cine*-substitution was not observed with any isomer of **14** under the reaction conditions chosen here. Therefore, the reaction conditions could be applied which were used for ^18^F-labelling of the three isomers of anisole ylide [28]. The required precursors were synthesised according to the same literature [28] and purified using column chromatography as proven successful.

**Figure 2:**
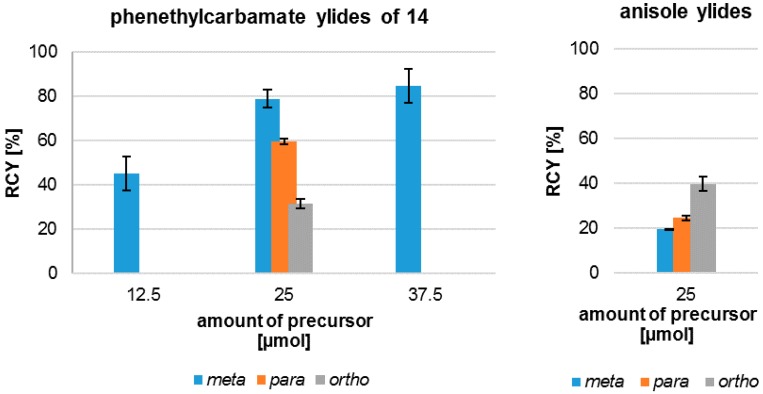
Effect of the amount of precursor on radiochemical yield (RCY) of different phenethylcarbamates and anisoles in dependence of the substitution pattern.

The radiofluorination of the examined iodonium ylides showed a high regioselectivity in all cases. In contrast to literature the formation of isomers was not observed. The yields of ^18^F-fluorinated products after systematic labelling of all isomers are shown in Figure 2. Furthermore, different radiochemical yields for the labelling of the three carbamate isomers are clearly depicted. The trend observed for radiochemical yield (RCY) (*meta* > *para* > *ortho*) depends on substitution pattern, mesomeric and inductive effects, but obviously also on a steric effect, considering the *ortho*-derivative.

Figure 2 also shows the relation between the amount of precursor **14m** used and the resulting radiochemical yield. A reduction of the mass of precursor to 12.5 µmol caused a decrease of the resulting RCY, while an increase to 37.5 µmol only modestly augmented the RCY and on account of a simultaneous enlargement of the standard deviation. An amount of 25 µmol seemed preferable in accordance with more constant product yields.

**Figure 3:**
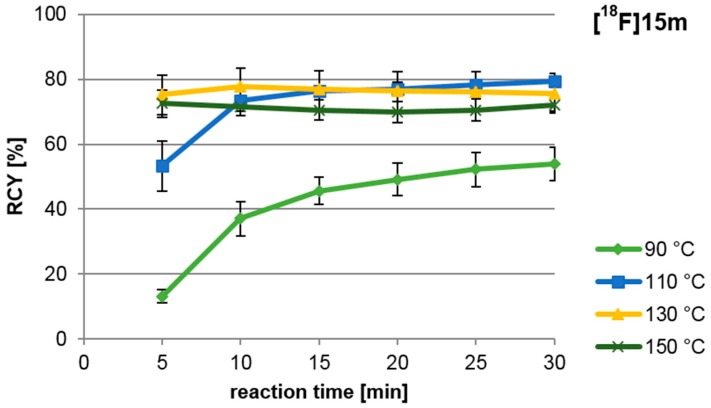
Influence of reaction temperature and time of the nucleophilic radiofluorination of ylide **14m**, yielding **[^18^F]15m**. For reagents and conditions see Scheme 4, step a.

A high yield of the labelled intermediate **[^18^F]15m** is to be achieved from **14m** for the “build-up” synthesis of the nNOS-tracer. Therefore, the dependence of the RCY of **[^18^F]15m** on reaction time and temperature was examined in detail (see Figure 3). Highest yields were obtained after 10 min at temperatures between 110 °C and 150 °C. At temperatures between 130 °C and 150 °C, however, a RCY of up to 73% was obtained already after a reaction time of 5 min.

For the following “build-up” synthesis an amount of precursor of 25 µmol and a reaction time of 10 min at 110 °C was chosen due to the comparably small standard deviations observed there.

**Scheme 4:**
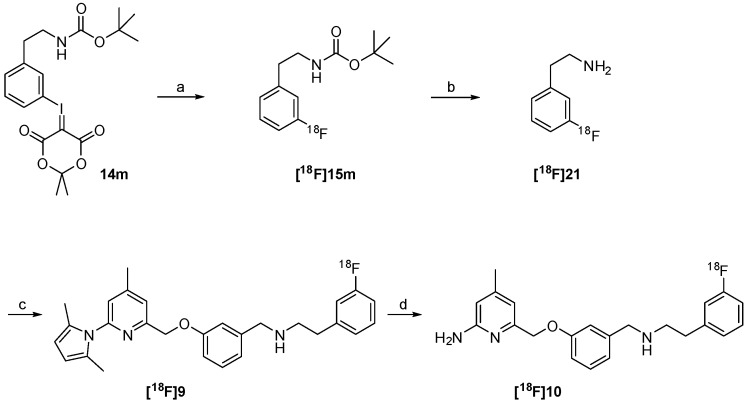
“Build-up” radiosynthesis of the nNOS-inhibitor **[^18^F]10**. *Reagents and conditions*: (a) Kryptofix^®^2.2.2, K_2_CO_3_, no-carrier-added (n.c.a.) [^18^F]fluoride, DMF, 110 °C, 10 min; (b) HCl in dioxane, 15 min, 80 °C; (c) **8**, NaBH(OAc)_3_, room temperature, 15 min; (d) NH_2_OH∙HCl, EtOH, H_2_O, max. 50 W microwave irradiation, 100 °C, 10 min.

The successful radiosynthesis of synthon **[^18^F]15m** provided a good basis for the following “build-up” synthesis of the nNOS-inhibitor. The intermediate **[^18^F]15m** was trapped after radiofluorination on a reversed-phase resin for removal of ionic components formed. 65%–80% of total radioactivity was retained on the column used in the ^18^F-fluorination process, whereas unreacted [^18^F]fluoride was most likely eluted as waste. After elution with dioxane and deprotection, the resulting amine **[^18^F]21** was converted by reductive amination in presence of the aldehyde **8** and sodium triacetoxyborohydride to the pyrrole protected nNOS-inhibitor **[^18^F]9** as shown in Scheme 4.

The reductive amination yielded compound **[^18^F]9** in a rather variable RCY of 6%–40% (*n* = 4). The subsequent deprotection was carried out under microwave heating. This four-step “build-up” process yielded the desired nNOS-inhibitor **[^18^F]10** in a maximum radiochemical yield of 15%. The procedure was not further optimized due to a highly promising “late-stage” ^18^F-labelling method examined in parallel.

### 2.3. Synthesis of Reference Compound and Precursor for a “Late-Stage” ^18^F-Labelling Procedure

The recently introduced “late-stage” approach for nucleophilic n.c.a. ^18^F-labelling, based on a boronic acid pinacol ester as precursor, seemed attractive for application to the radiofluorination of the desired nNOS tracer. Especially, the new copper-mediated method for labelling organic boronic acid esters with n.c.a. [^18^F]fluoride as published in 2014 by Tredwell et al. [30] found ample application in drug development and got into the focus of radiochemistry. The radiofluorination of electron-rich arenes, generally not amenable to aromatic nucleophilic substitution (S_N_Ar) with [^18^F]fluoride, can thereby be performed through reaction of pinacol-derived aryl boronic esters with [^18^F]KF/K_222_ in the presence of [Cu(OTf)_2_(py)_4_] (OTf = trifluoromethanesulfonate, py = pyridine). This method was meanwhile adapted to various aromatic systems, as it tolerates a variety of different functional groups [30,31]. Therefore, it is of high interest, and it appeared potentially useful as alternative for labelling of the desired nNOS inhibitor.

**Scheme 5:**
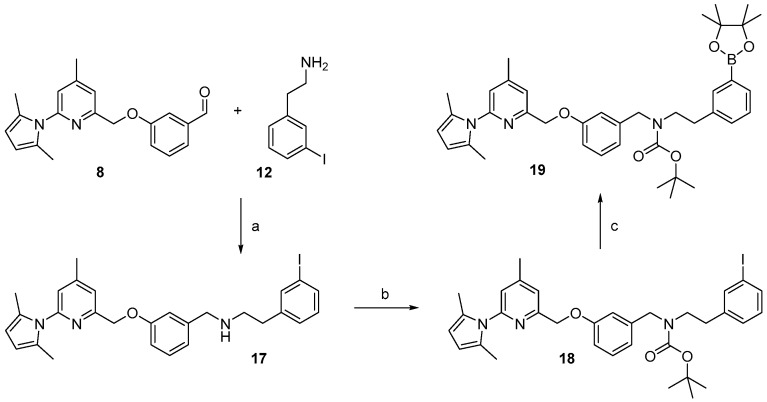
Synthesis of the boronic acid pinacol ester, precursor **19**. *Reagents and conditions*: (a) NaBH(OAc)_3_, MeOH, 3 h, room temperature; (b) Boc_2_O, DMAP, MeOH, 15 min, room temperature; (c) *bis*-pinacolatodiborane, potassium acetate, [1,1′-bis(diphenylphosphino)ferrocene]dichloropalladium(II), DMF, 85 °C, 90 min.

For the preparation of a corresponding boronic acid pinacol ester precursor the appropriate iodinated derivative **18** of the nNOS-inhibitor was synthesized as starting material. This was achieved by reductive amination of aldehyde **8** with 3-iodophenethylamine **12** (see Scheme 5).

**Scheme 6:**
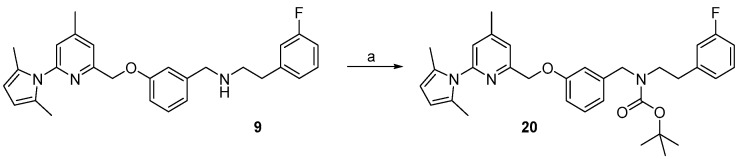
Synthesis of the reference compound of intermediate **20**. Reagents and conditions: (a) Boc_2_O, DMAP, MeOH, 15 min, room temperature.

For the cross-coupling as well as for the radiosynthesis, the secondary amine **17** was Boc-protected in high yield of about 87%. The pyrrole- and Boc-protected iodide **18** was converted by a palladium catalysed cross-coupling reaction to the pinacol ester **19** in ca. 64% yield. For the work-up process of **19** the iodide **18** had to be fully converted, otherwise the product could not be separated from the starting material. Therefore, an extensive work-up procedure, including preparative reversed-phase column chromatography, was needed, since normal-phase chromatography led to decomposition of **19**. The Boc-protected ^19^F-reference compound **20** was synthesized to unambiguously identify the radiolabelled product, starting from **9** in about 83% RCY (see Scheme 6).

### 2.4. Radiosynthesis of the nNOS-Inhibitor ***[^18^F]10*** by “Late-Stage” Nucleophilic ^18^F-Labelling

The preparation of the nNOS-inhibitor **[^18^F]10** started with the ^18^F-fluorination of the boronic ester (ArylBPin) **19** in the presence of n.c.a. [^18^F]fluoride and the copper(II)-complex [Cu(OTf)_2_(py)_4_] for mediation of the [^18^F]fluorine-aryl bond formation. In accordance to literature [31] it was found, that a conventional labelling protocol, using macroscopic amounts of [^18^F]KF/K_222_ in acetonitrile, is not applicable for this copper-mediated labelling method. The original labelling protocol [30] also indicates that only comparatively low amounts of potassium carbonate and Kryptofix^®^2.2.2 will allow product formation.

With regard to a prospective automation, a one-pot procedure for the preparation of the nNOS-inhibitor was developed. Drying and activating of the [^18^F]fluoride ion with potassium carbonate and Kryptofix^®^2.2.2 as well as copper mediated labelling should ideally be performed in one reaction vessel. A diminution of the amounts of potassium carbonate and Kryptofix^®^2.2.2 is desirable, but also limited due to a decreasing efficiency of [^18^F]fluoride release using QMA cartridges. Hence, a minimum amount of anions is necessary for an efficient release of [^18^F]fluoride from QMA cartridge after fixation.

The best results were observed with 46 mg QMA-cartridges from Waters^®^ (Milford, MA, USA) and an elution mixture consisting of 2.6 µmol of tetraethylammonium bicarbonate (TEAHCO_3_) in 0.8 mL of methanol as a compromise between efficient [^18^F]fluoride release from the resin and moderate ^18^F-labelling yields. Under these conditions the loss of [^18^F]fluoride during fixation and release, which was carried out in the same flow direction, did not exceed 15%.

**Scheme 7:**
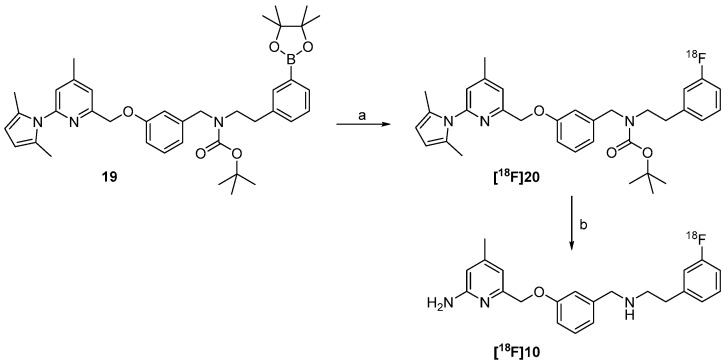
“Late-stage” ^18^F-labelling of the nNOS-inhibitor **[^18^F]10**. *Reagents and conditions*: (a) TEAHCO_3_, n.c.a. [^18^F]fluoride, DMF, 110 °C, 15 min; (b) NH_2_OH∙HCl, EtOH, H_2_O, max. 50 W microwave irradiation, 100 °C, 10 min.

In accordance to the literature and along with the proposed reaction mechanism of the copper mediated fluorination [32], it was found that the ventilation of the Wheaton^®^ (Millville, NJ, USA) vial with air after the drying process has a positive effect on the RCY. After the drying procedure the precursor was converted in presence of the copper-complex to **[^18^F]20** (see Scheme 7). The influence of the reaction temperature and time on this radiofluorination was also examined in more detail. Previous studies on the copper-mediated reaction of [^18^F]fluoride with pinacol boronic esters indicated that the use of DMF as solvent and a temperature of 110 °C gave high RCYs [30,31].

**Figure 4:**
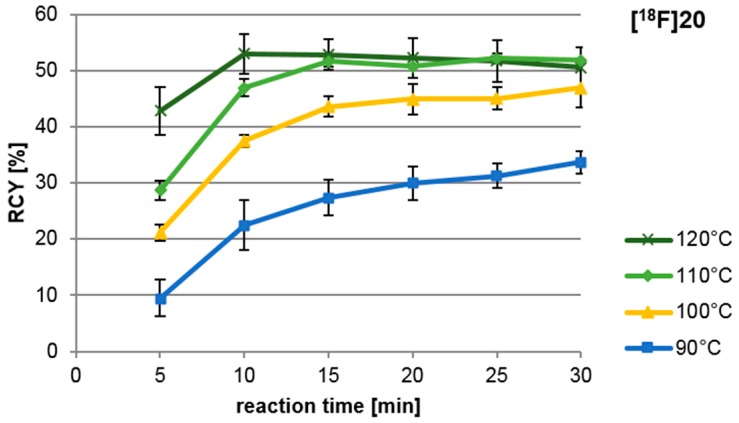
Influence of reaction temperature and time on copper-mediated radiofluorination of precursor **19**. All syntheses were carried out manually and RCY was determined by radio thin layer chromatography (TLC). Each experiment was carried out at least in triplicate. Reagents and conditions: 30 µL MeCN, 10 mg (15 µmol) **19**, 3.5 mg (5 µmol) Cu^II^OTf_2_py_4_ in 300 µL DMF.

Figure 4 shows that the highest yields were obtained after 10 min reaction time at 120 °C; however, standard deviations are comparatively high. More reproducible results were achieved after 15 min at 110 °C. Higher temperatures and longer reaction times led to the formation of polar, radioactive by-products. These unidentified products though, never exceeded 5% of the total activity. Reaction times longer than 15 min seem only relevant if low temperatures are necessary with given sensible precursors.

The ^18^F-fluorinated product **[^18^F]20** was trapped on a reversed-phase cartridge and eluted for deprotection of the pyrrole and the Boc-group into a special microwave vessel. After microwave treatment in the presence of NH_2_OH·HCl the product was easily isolated by solid phase extraction. The radiochemical yield of the desired nNOS-tracer **[^18^F]10** based on the starting activity was about 14% ± 5% (*n* = 4). Even with the small amounts of n.c.a. [^18^F]fluoride (30–100 MBq) used for the developmental work performed here, high molar activities of ≥48 GBq/µmol were achieved. Batch production of ^18^F-labelled ligands with higher starting activity, carried out with the same technological equipment in our institute, however, resulted reliably in even higher molar activities of ≥90 GBq/µmol [33].

## 3. Materials and Methods

### 3.1. General Information

All chemicals were purchased from Sigma-Aldrich GmbH (Steinheim, Germany), Fluka (Buchs, Switzerland), Merck KGaA (Darmstadt, Germany), ChemPUR GmbH (Karlsruhe, Germany) or Activate Scientific (Prien, Germany) and used without further purification. All reactions were carried out by standard air-free and moisture-free techniques under an inert atmosphere of argon. Dry solvents were purchased in sufficient purity.

Thin layer chromatography (TLC) was done on precoated plates ALUGRAM^®^ SIL G/UV_254_ (Macherey-Nagel GmbH, Düren, Germany) or on TLC silica gel 60 RP-18 F_254_**S** (Merck KGaA), and the compounds were detected at 254 nm. Flash column chromatography was conducted manually on silica gel 60 (35–70 μm) from Merck KGaA or by using the Reveleris™ flash chromatography system from Grace (Worms, Germany) with evaporative light scattering (ELS) and UV detection. ^1^H-, ^11^B-, ^13^C- and ^19^F-Nuclear Magnetic Resonance (NMR) spectra were recorded at ZEA (Forschungszentrum Jülich) on a Bruker DPX Avance 200 spectrometer (Bruker, Billerica, MA, USA), a Varian Inova 400 MHz (Varian Inc., Palo Alto, CA, USA) or on a Bruker Avance 600. All shifts are given below in δ (ppm) using the signals of the corresponding solvent as reference. All coupling constants (*J*) are given in Hz. Splitting patterns are typically described as follows: s: singlet, d: doublet, t: triplet, m: multiplet. Mass spectra were obtained with a Finnigan Automass Multi mass spectrometer with an electron beam energy of 70 eV. High-resolution mass spectrometry (HRMS) spectra were recorded on a FTICR “LTQ FT Ultra” (both from Thermo Fisher Scientific, Braunschweig, Germany). Elemental analyses (EA, microanalyses) were carried out at ZEA (Forschungszentrum Jülich) on a Vario EL cube, elemental analyser (Elementar, Hanau, Germany). Experiments under microwave heating were performed using a CEM Discover (Matthews, NC, USA) single-mode microwave reactor system.

Radioactivity on radio-TLC plates was detected on an InstantIMAGER and associated software (Packard, Dreieich, Germany). Sep-Pak^®^ C-18 plus-cartridges were purchased from Waters^®^ (Eschborn, Germany) and conditioned with 10 mL of ethanol and 10 mL water, while Sep-Pak^®^ Light QMA Carb-cartridges, also purchased from Waters^®^, were conditioned using 10 mL of water only.

Chromatographic systems: High-performance liquid chromatography (HPLC) was performed on the following system from Dionex (Idstein, Germany): an Ultimate 3000 LPG-3400A HPLC pump, an Ultimate 3000 VWD-3100 UV/VIS-detector, a UCI-50 chromatography interface, an injection valve P/N 8215 and a NaI(Tl) well-type scintillation detector (Raytest, Straubenhardt, Germany) and associated electronics for radioactivity detection. Data acquisition and interpretation were performed with Chromeleon software (Thermo Scientific Dionex™, Version 6.8). Isocratic separations were carried out on a Knauer^®^ HPLC-system comprising a Knauer Azura P 4.1S pump, a Knauer Azura UVD 2.1S UV/VIS-detector (Knauer, Berlin, Germany), a Rheodyne injector (20 µL loop), and a NaI(Tl) well-type scintillation detector (EG&G Ortec; model 276 Photomultiplier Base) with an ACE Mate Amplifier and BIAS supply (all from Ortek Ametek, Meerbusch, Germany) for radioactivity detection. Data acquisition and interpretation were performed with Gina software (Raytest, Version 5.9 SP10).

Analytical HPLC was performed with the following columns:

a: Gemini 5 μm C18 110 Å with appropriate Gemini C18 Security Guard 250 mm × 4.6 mm (Phenomenex, Aschaffenburg, Germany)
b: Luna 5 μm C18(2) 100 Å with appropriate Luna C18 Security Guard 250 mm × 4.6 mm (Phenomenex)
c: Luna 5 μm PFP(2) 100 Å with appropriate Luna PFP(2) Security Guard 250 mm × 4.6 mm (Phenomenex)

In all cases elution was performed at a constant flow rate of 1 mL∙min^−1^. UV and radioactivity detectors were connected in series, corresponding to a time delay of 0.5–0.9 min depending on the flow rate. ^18^F-Labelled compounds were identified by spiking the reaction mixture with corresponding ^19^F-reference compounds, using HPLC. The capacity factor values (*k*-values) of important radiolabelled compounds together with the corresponding separation systems are summarized in Table 1 and Table 2. The *R*_f_-values of important radiolabelled compounds and the corresponding TLC conditions are summarized in Table 3.

### 3.2. Radiochemistry

No-carrier-added [^18^F]fluoride was produced via the ^18^O(p,n)^18^F nuclear reaction by bombardment of an isotopically enriched [^18^O]water target with 17 MeV protons at the JWS cyclotron BC 1710 in house [34]. For all radiosyntheses 5 mL V-vials (Wheaton) were used. A Curiementor 2 dose calibrator (PTW GmbH, Freiburg, Germany) was used for activity measurements. For all radio-HPLC analyses (determination of RCY), activity was quantified by injection of a reference aliquot on a second injection port behind the column, to measure the total activity of the aliquot (corrected for decay).

#### 3.2.1. Radiosynthesis of *tert*-butyl-([^18^F]fluorophenethyl)carbamate (**[^18^F]15**)

The radiosyntheses during the developmental studies were typically performed with 30–60 MBq n.c.a. [^18^F]fluoride which was preprocessed by standard procedures [28]. Differently, however, the amounts of potassium carbonate and acetonitrile used were higher with 0.019 mmol and 1 mL, respectively. After purging the reaction vessel with argon, a solution of 12 mg (25 µmol) of labelling precursor **14** in 1 mL of dry DMF was added into the reaction vessel. During the reaction aliquots of 20 µL were taken at various temperatures and quenched in 0.4 mL of water and acetonitrile (1:1 *v/v*). Each mixture was analysed in triplicate by radio-TLC. The radiochemical yield was calculated from the TLC-radiochromatogram and is defined as the ratio of radioactivity area of the desired product **15** (SiO_2_-TLC, EtOAc/PE 1:4 *v/v*, *R*_f_ 0.5) over the total area of fluorine-18 radioactivity which was placed above the solvent front on the TLC-plate. For the examination of regioselectivity an aliquot of 500 µL was removed for deprotection in presence of 100 µL of a 1 M HCl solution. After 10 min at 90 °C the reaction vessel was cooled in an ice bath. The mixture was titrated with 1 M NaOH solution back on pH 9 before an aliquot was taken for radio-HPLC measurements.

#### 3.2.2. Radiosynthesis of 2-, 3- and 4-[^18^F]fluoroanisole

Typically 30–60 MBq of n.c.a. [^18^F]fluoride was prepared as described above for the radiosyntheses of isomeric [^18^F]fluoroanisoles from corresponding ylide precursors, which was carried out as described in the literature [29]. After completing the reaction, an aliquot was taken for radio-HPLC measurement and for determination of regioselectivity.

#### 3.2.3. Radiosynthesis of **[^18^F]10** via the “Build-up” Synthesis

For the “build-up” synthesis of **[^18^F]10** typically 200 MBq of [^18^F]fluoride was azeotropically dried as described in 3.2.1 and the reaction vessel was purged with argon. A solution of 12 mg (25 µmol) labelling precursor **14m** and 1 mL of dry DMF was transferred into the V-vial. The mixture was stirred at 110 °C for 10 min. After radiolabelling, the V-vial was cooled down in an ice bath and radioactivity was measured. The mixture was diluted in 10 mL water, and the reaction vessel was rinsed with 0.3 mL DMF. The combined mixtures were transferred through a Sep-Pak^®^ Plus C18 cartridge which was previously conditioned with 10 mL of EtOH and 20 mL of water. Additionally 10 mL of water were passed through the cartridge before 40 mL of air were purged through.

The intermediate compound **[^18^F]15m** was then eluted with 0.9 mL of dioxane from the reversed phase resin into a new V-vial. For the deprotection 0.1 mL HCl-dioxane (4 M) were added, and the resulting mixture was stirred at room temperature for 2 min. After 15 min at 80 °C and 70 mbar under a stream of argon the mixture was cooled down to room temperature. Then 0.5 mL water and 0.1 mL of an aqueous NaOH-solution (4 M) were added. The corresponding amine **[^18^F]21** was extracted three times with 1.0 mL of dichloromethane. The combined organic layers were dried by a Na_2_SO_4_-cartridge (0.9 cm in diameter and 4.5 cm in length) and transferred into a new V-vial.

After evaporation of the solvent under reduced pressure the amine was converted by reductive amination in presence of the aldehyde **8** (30 mg, 0.09 mmol) and sodium triacetoxyborohydride (30 mg, 0.14 mmol) in 0.5 mL of methanol to the pyrrole protected nNOS-inhibitor **[^18^F]9**. After 15 min at room temperature the mixture was concentrated and alkalised with 0.1 mL of NaOH solution (4 M). The product **[^18^F]9** was extracted again with dichloromethane. The combined organic layers were transferred into a microwave vessel and concentrated at 40 °C under a stream of argon. A solution of 345 mg NH_2_OH∙HCl in 1 mL EtOH and 0.5 mL water was then added for the deprotection of the pyrrole. This was carried out by microwave heating for 10 min at 100 °C (max. 50 W). Eventually, an aliquot of the irradiated product was alkalised and analysed by radio-HPLC and radio-TLC. This procedure yielded the desired nNOS-inhibitor **[^18^F]10** in pure form.

#### 3.2.4. Radiosynthesis of **[^18^F]10** via “Late-Stage” Labelling

Different to the procedure described in Section 3.2.1. [^18^F]fluoride was preprocessed for radiosynthesis as follows: an aliquot of approx. 100 MBq n.c.a. [^18^F]fluoride in 500 µL of water was purged through a 46 mg QMA-cartridge (Waters^®^, Sep-Pak Accell Plus QMA Carbonate Plus Light). After rinsing the cartridge with 1 mL of methanol a solution of 2.6 µmol of TEAHCO_3_ in 0.8 mL of methanol was slowly (1 min) passed through the resin into a V-vial. The vial was equipped with a cannula and sterile filter. At 80 °C and 250 mbar the solvent was removed in 5 min under a stream of air. Afterwards, the cannula was removed and the pressure was lowered to 6–12 mbar for 5 min. The vial was then ventilated by the cannula again and cooled to RT. For radiolabelling a solution of 30 µL of acetonitrile, 10 mg (15 µmol) of precursor 19 and 3.5 mg (5 µmol) of Cu^II^OTf_2_py_4_ in 300 µL of DMF was added. The mixture was stirred for 10 min at 120 °C. During the reaction the cannula was not removed.

After radiofluorination the mixture was completely transferred into 50 mL of water (reactor was rinsed with 0.5 mL of ethanol). The aqueous product mixture was then rinsed through a C18 cartridge (Waters^®^, SepPak, 500 mg), and 10 mL of water and 40 mL of air were purged through the resin. The product was eluted from the resin into a microwave vessel with 3 mL of ethanol. A solution of 345 mg of NH_2_OH∙HCl in 1 mL water was added. Subsequent deprotection was carried out by microwave heating for 10 min at 100 °C (max. 50 W), and then the mixture alkalised with a NaOH-solution (pH >9) and diluted with 50 mL of water containing 0.5 wt % of Na_3_PO_4_∙12H_2_O (titrated with concentrated H_3_PO_4_ to pH 8.5). The product was trapped on the C18 cartridge (Waters^®^, SepPak, 500 mg) by passing the aqueous mixture though the resin. 10 mL of water and 40 mL air were purged through it before elution of the product with 3 mL acetonitrile, yielding the desired nNOS-tracer **[^18^F]10** in radiochemically pure form. The molar activity of **[^18^F]10** after preparation by both methods was measured by determination of product mass and activity, using the HPLC system ‘Dionex’ and condition ‘7’ as listed above in Table 1 and Table 2, respectively.

### 3.3. Precursor Syntheses

#### 3.3.1. Synthesis of Iodonium Ylide Precursors

##### General procedure for the Reduction of Nitriles

The compounds **22o**, **22m** and **22p** were prepared as described in the literature [35,36].

*2-Iodophenylethylamine hydrochloride* (**12o**)


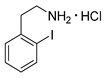


Analytical data correspond to the reported spectroscopic values in literature [36].

*3-Iodophenylethylamine hydrochloride* (**12m**)


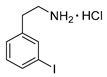


^1^H-NMR (200.13 MHz, DMSO-*d*_6_) δ 8.30 (b, 3H), 7.64 (m, 2H), 7.31 (d, ^3^*J* = 7.5 Hz, 1H), 7.14 (t, ^3^*J* = 7.5 Hz, 1H), 2.91–3.06 (m, 4H). ^13^C-NMR (50.33 MHz, DMSO-*d*_6_) δ 140.7, 137.7, 135.9, 131.2, 128.7, 95.7, 40.1, 32.8. HRMS (C_8_H_10_IN, [M + H]^+^) calcd: 247.9931, found: 247.9931. EA calcd: C (33.89), H (3.91), N (4.94), found: C (34.14 ± 0.13), H (3.95 ± 0.02), N (4.78 ± 0.02).

*4-Iodophenylethylamine hydrochloride* (**12p**)


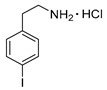


^1^H-NMR (600.15 MHz, DMSO-*d*_6_) δ 8.16 (b, 3H), 7.68 (d, ^3^*J* = 6.0 Hz, 2H), 7.10 (d, ^3^*J* = 6.0 Hz, 2H), 3.00 (t, ^3^*J* = 6.5 Hz, 2H), 2.87 (t, ^3^*J* = 6.5 Hz, 2H). ^13^C-NMR (150.92 MHz, DMSO-*d*_6_) δ 137.8, 137.7, 131.6, 93.1, 40.0, 32.9. HRMS (C_8_H_10_IN, [M + H]^+^) calcd: 247.9931, found: 247.9930.

##### General Procedure for Boc-Protection of **13**

A mixture of 283 mg (1 mmol) iodophenethylamine hydrochloride, 218 mg (1 mmol) Boc_2_O and 250 mg (3 mmol) NaHCO_3_ in 5 mL methanol was treated with ultrasound for 30 min at 50 °C. After filtration and removal of the solvent under reduced pressure, the crude product was purified by flash chromatography (petroleum ether/20% ethyl acetate, *R*_f_ = 0.6 (*ortho*, *para*), *R*_f_ = 0.5 (*meta*)) to obtain the product as a colourless oil (yields: **13o**: 306 mg, 0.88 mmol, 88%; **13m**: 326 mg, 0.94 mmol, 94%; **13p**: 333 mg, 0.96 mmol, 96%).

*tert-Butyl-(2-iodophenethyl)carbamate* (**13o**)


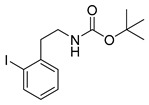


Analytical data correspond to the reported spectroscopic values in literature [36].

*tert-Butyl-(3-iodophenylethyl)carbamate* (**13m**) [37]


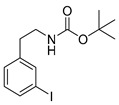


^1^H-NMR (200.13 MHz, CDCl_3_) δ 7.61–7.58 (m, 2H), 7.21–7.03 (m, 2H), 4.66 (b, 1H), 3.38 (t, ^3^*J* = 6.7 Hz, 2H), 2.77 (t, ^3^*J* = 7.0 Hz, 2H), 1.48 (s, 9H). ^13^C-NMR (50.33 MHz, CDCl_3_) δ 155.8, 141.5, 137.8, 135.5, 130.3, 128.1, 94.6, 79.3, 42.1, 35.8, 28.4. HRMS (C_13_H_18_INO_2_) calcd: 348.0455 [M + H]^+^, 370.0291 [M + Na]^+^, found: 348.0451[M + H]^+^, 370.0282 [M + Na]^+^. EA calcd: C (44.97), H (5.23), N (4.03), found: C (44.64 ± 0.11), H (5.18 ± 0.02), N (3.65 ± 0.08).

*tert-Butyl-(4-iodophenethyl)carbamate* (**13p**)


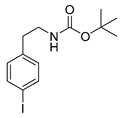


^1^H-NMR (200.13 MHz, CDCl_3_) δ 7.64 (dt, ^3^*J* = 8.4 Hz, ^4^*J* = 1.9 Hz, 2H), 7.32 (dt, ^3^*J* = 8.4 Hz, ^4^*J* = 1.9 Hz, 2H), 4.45 (b, 1H), 3.37 (t, ^3^*J* = 6.9 Hz, 2H), 2.77 (t, ^3^*J* = 7.0 Hz, 2H), 1.46 (s, 9H). ^13^C-NMR (50.32 MHz, CDCl_3_) δ 155.9, 138.7, 137.6, 130.9, 91.6, 79.4, 41.8, 35.8, 28.4. HRMS (C_13_H_18_INO_2_) calcd: 348.0455 [M + H]^+^, 370.0274 [M + Na]^+^, found 348.0467 [M + H]^+^, 370.0291 [M + Na]^+^. EA calcd: C (44.97), H (5.23), N (4.03), found: C (45.29 ± 0.04), H (5.24 ± 0.01), N (3.75 ± 0.03).

*tert-Butyl-(fluorophenethyl)carbamate* (**15**)

A stirred mixture of 139 mg (1 mmol) fluorophenethylamine and 218 mg (1 mmol) Boc_2_O in 2 mL methanol was treated with ultrasound for 30 min at 50 °C. After removal of the solvent under reduced pressure the crude product was purified by flash chromatography (petroleum ether/20% ethyl acetate, *R*_f_ = 0.6 (*ortho*, *para*), 0.5 (*meta*)) to obtain the product as a colourless oil (yields: **15o**: 232 mg, 0.97 mmol, 97%; **15m**: 234 mg, 0.98 mmol, 98%; **15p**: 229 mg, 0.96 mmol, 96%).

*tert-Butyl-(2-fluorophenethyl)carbamate* (**15o**)


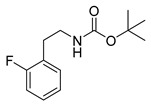


^1^H-NMR (200.13 MHz, CDCl_3_) δ 7.00–7.30 (m, 4H), 4.70 (b, 1H), 3.40 (t, *^3^J* = 7.0 Hz, 2H), 2.87 (t, *^3^J* = 7.0 Hz, 2H), 1.46 (s, 9H). ^13^C-NMR (50.33 MHz, CDCl_3_) δ 161.3 (d, ^1^*J*_CF_ = 245 Hz), 155.9, 131.2 (d, ^3^*J*_CF_ = 5 Hz), 128.2 (d, ^3^*J*_CF_ = 8 Hz), 125.9 (^2^*J*_CF_ = 16 Hz), 124.1 (d, ^4^*J*_CF_ = 4 Hz), 115.3 (d, ^2^*J*_CF_ = 22 Hz), 79.3, 40.8, 29.8, 28.4. ^19^F-NMR (188.31 MHz, CDCl_3_) δ −118.6. HRMS (C_13_H_18_FNO_2_) calcd: 240.1394 [M + H]^+^, 370.0291 [M + Na]^+^, found: 240.1393 [M + H]^+^, 262.1214 [M + Na]^+^. EA calcd: C (65.25), H (7.58), N (5.85), found: C (65.03 ± 0.04), H (7.62 ± 0.06), N (5.85 ± 0.05).

*tert-Butyl-(3-fluorophenethyl)carbamate* (**15m**)


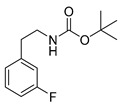


UV-absorption (λ_max_, nm) 210, 261. ^1^H-NMR (200.13 MHz, CDCl_3_) δ 7.23–7.20 (m, 1H), 6.99–6.87 (m, 3H), 4.75 (b, 1H), 3.36 (t, ^3^*J* = 6.9 Hz), 2.79 (t, ^3^*J* = 7.0 Hz), 1.44 (s, 9H). ^13^C-NMR (50.33 MHz, CDCl_3_) δ 162.5 (d, ^1^*J_CF_* = 246 Hz), 155.9, 141.6 (d, ^3^*J*_CF_ = 7 Hz), 129.9 (d, ^3^*J*_CF_ = 8 Hz), 124.4 (d, ^4^*J*_CF_ = 3 Hz), 115.6 (d, ^2^*J*_CF_ = 21 Hz), 113.2 (d, ^2^*J*_CF_ = 21 Hz), 79.3, 41.8, 36.0, 28.4. ^19^F-NMR (188.31 MHz, CDCl_3_) −113.4. HRMS (C_13_H_18_FNNaO_2_) calcd: 262.1214 [M + Na]^+^, found: 262.1222 [M + Na]^+^. EA calcd: C (65.25), H (7.58), N (5.85), found: C (65.24 ± 0.10), H (7.55 ± 0.01), N (5.78 ± 0.03).

*tert-Butyl-(4-fluorophenethyl)carbamate* (**15p**)


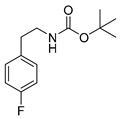


^1^H-NMR (200.13 MHz, CDCl_3_) δ 7.16 (t, ^3^*J* = 6.0 Hz, 2H), 7.01 (t, ^3^*J* = 6.6 Hz, 2H), 4.60 (b, 1H), 3.36 (t, ^3^*J* = 7.0 Hz, 2H), 2.79 (t, ^3^*J* = 7.0 Hz, 2H), 1.46 (s, 9H). ^13^C-NMR (50.32 MHz, CDCl_3_) δ 161.6 (d, ^1^*J*_CF_ = 244 Hz), 155.9, 134.7 (d, ^4^*J*_CF_ = 3 Hz), 130.2 (d, ^3^*J*_CF_ = 8 Hz), 115.3 (d, ^2^*J*_CF_ = 21 Hz), 113.2 (d, ^2^*J*_CF_ = 21 Hz), 79.3, 42.1, 35.5, 28.4. ^19^F-NMR (188.31 MHz, CDCl_3_) δ −117.4. HRMS (C_13_H_18_FNNaO_2_) calcd: 262.1214 [M + Na]^+^, found: 262.1215 [M + Na]^+^. EA calcd: C (65.25), H (7.58), N (5.85), found: C (65.29 ± 0.19), H (7.65 ± 0.03), N (5.78 ± 0.02).

##### General Procedure for the Synthesis of Iodonium Ylides from Aryliodides

In a 50 mL round bottom flask, 1.00 g (2.88 mmol) *tert*-butyl(iodophenethyl)carbamate and 662 mg (2.88 mmol) of *m*-CPBA were suspended in 8 mL of DCM and stirred for 2 h at room temperature. In a separate vessel 498 mg (3.45 mmol) of Meldrum’s acid and 960 mg (17.11 mmol) of KOH were stirred in 8 mL of DCM for 15 min. Then the mixture containing the oxidized iodobenzene was added to the potassium salt of Meldrum’s acid. Remaining solids were suspended in 5 mL of DCM and also added. After 3 h stirring at RT the mixture was filtered over cellulose, the filter washed with 40 mL of DCM and the filtrates concentrated under reduced pressure. The crude product was purified by flash chromatography with ethyl acetate and acetone (1:1 *v/v*) for the *meta*-derivative (**14m** *R*_f_ = 0.5) or with 100% ethyl acetate for the *ortho*- and *para*-isomers (**14o** *R*_f_ = 0.4 and **14p** *R*_f_ = 0.2), to obtain the product as a slightly yellow solid (yields: **14o**: 648 mg, 1.32 mmol, 46%; **14m**: 606 mg, 1.24 mmol, 43%; **14p**: 634 mg, 1.30 mmol, 45%).

*2-tert-Butoxycarbonylaminoethylphenyl-iodonium-(5-[2,2-dimethyl-1,3-dioxane-4,6-dione]) ylide* (**14o**)


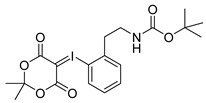


^1^H-NMR (600.15 MHz, DMSO-*d*_6_) δ 7.95 (d, ^3^*J* = 6.0 Hz), 7.52 (t, ^3^*J* = 6.0 Hz, 1H), 7.42 (d, ^3^*J* = 6.0 Hz, 1H), 7.26 (t, ^3^*J* = 6.0 Hz, 1H), 3.28 (t, ^3^*J* = 6.0 Hz, 1H), 3.06 (t, ^3^*J* = 6.0 Hz, 1H), 1.52 (s, 6H), 1.37 (s, 9H). ^13^C-NMR (150.92 MHz, DMSO-*d*_6_) δ 163.2, 156.1, 141.4, 136.6, 132.0, 130.7, 129.5, 122.6, 103.1, 78.2, 59.2, 41.0, 38.5, 28.4, 25.7. HRMS (C_19_H_24_INO_6_) calcd: 512.0541 [M + Na]^+^, 528.0280 [M + K]^+^, found 512.0547 [M + Na]^+^, 528.0286 [M + K]^+^. EA calcd: C (46.64), H (4.94), N (2.86), found: C (47.20 ± 0.10), H (5.48 ± 0.02), N (2.75 ± 0.03).

*3-tert-Butoxycarbonylaminoethylphenyl-iodonium-(5-[2,2-dimethyl-1,3-dioxane-4,6-dione]) ylide* (**14m**)


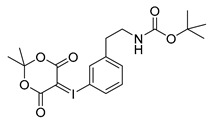


^1^H-NMR (200.13 MHz, DMSO-*d*_6_) δ 7.68–7.62 (m, 2H), 7.40–7.37 (m, 2H), 3.16 (t, ^3^*J* = 6.7 Hz, 2H), 2.73 (t, ^3^*J* = 6.9 Hz, 2H), 1.59 (s, 6H), 1.38 (s, 9H). ^13^C-NMR (50.33 MHz, DMSO-*d*_6_) δ 163.3, 156.0, 143.1, 133.0, 131.3, 130.7, 116.8, 103.2, 102.5, 78.1, 58.2, 41.6, 35.6, 28.4, 25.8. HRMS (C_19_H_24_INO_6_) calcd: 490.0721 [M + H]^+^, 528.0280 [M + K]^+^, found: 490.0724 [M + H]^+^, 528.0282 [M + K]^+^. EA calcd: C (46.64), H (4.94), N (2.86), found: C (46.10 ± 0.09), H (5.05 ± 0.04), N (3.06 ± 0.05).

*4-tert-Butoxycarbonylaminoethylphenyl-iodonium-(5-[2,2-dimethyl-1,3-dioxane-4,6-dione]) ylide* (**14p**)


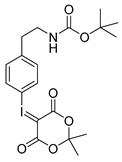


^1^H-NMR (200.13 MHz, DMSO-*d*_6_) δ 7.73 (d, ^3^*J* = 8.4 Hz, 2H), 7.28 (d, ^3^*J* = 8.4 Hz, 2H), 3.17 (t, ^3^*J* = 6.8 Hz, 2H), 2.73 (t, ^3^*J* = 6.9 Hz, 2H), 1.58 (s, 6H), 1.36 (s, 9H). ^13^C-NMR (50.33 MHz, DMSO-*d*_6_) δ 163.4, 156.1, 143.2, 133.1, 131.7, 113.2, 103.3, 78.1, 58.3, 41.3, 35.4, 28.5, 25.9. HRMS (C_19_H_24_INO_6_) calcd: 490.0721 [M + H]^+^, 512.0541 [M + Na]^+^, found: 490.0721 [M + H]^+^, 512.0592 [M + Na]^+^. EA calcd: C (46.64), H (4.94), N (2.86). Found: C (46.59 ± 0.05), H (5.24 ± 0.02), N (2.98 ± 0.02). 

In case of the isomeric iodonium ylides of anisole 1.5 g (6.45 mmol) of the corresponding iodoanisole was used as starting amount, following otherwise the general procedure given above (yields: *ortho*-anisole ylide: 1.070 g, 2.84 mmol, 44%; *meta*-anisole ylide: 1.010 g, 2.68 mmol, 42%; *para*-anisole ylide: 978 mg, 2.60 mmol, 41%).

2-Methoxyphenyl-iodonium-(5-[2,2-dimethyl-1,3-dioxane-4,6-dione]) ylide


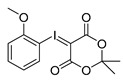


Analytical data correspond to the reported spectroscopic values in literature [29].

3-Methoxyphenyl-iodonium-(5-[2,2-dimethyl-1,3-dioxane-4,6-dione]) ylide


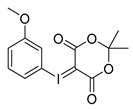


Analytical data correspond to the reported spectroscopic values in literature [29].

4-Methoxyphenyl-iodonium-(5-[2,2-dimethyl-1,3-dioxane-4,6-dione]) ylide


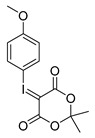


Analytical data correspond to the reported spectroscopic values in literature [29].

#### 3.3.2. Synthesis of Reference Compounds and Iodinated Intermediates for the Preparation of the Boronic Ester Precursor

Compounds **4**, **6**, **7** and **8** were prepared as described in the literature [25].

*N-(3-((6-(2,5-Dimethyl-1H-pyrrol-1-yl)-4-methylpyridin-2-yl)methoxy)benzyl)-2-(3-fluorophenyl)ethanamine* (**9**)

To a solution of 153 mg (1.1 mmol) of 3-fluorophenethyl amine in 4 mL of dry methanol 320 mg (1 mmol) of 3-((6-(2,5-dimethyl-1*H*-pyrrol-1-yl)-4-methylpyridin-2-yl)methoxy)-benzaldehyde in 2 mL of dry methanol were added dropwise. Then 424 mg (2 mmol) of NaBH(OAc)_3_ were added carefully, and the mixture was allowed to react at room temperature for 1 h. After filtration and removal of the solvent, the residue was diluted with 15 mL of 1 N NaOH and extracted with CH_2_Cl_2_ (3 × 10 mL). The organic layers were combined, washed with brine, dried over NaSO4, and concentrated under reduced pressure to give the crude product, which was purified by flash chromatography (ethyl acetate/9% petroleum ether/1% triethyl amine) to receive a pale yellow oil in 89% yield (395 mg, 0.89 mmol).


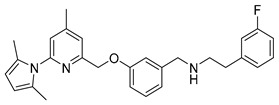


Analytical data correspond to the reported spectroscopic values in literature [25].

*N-(3-((6-(2,5-Dimethyl-1H-pyrrol-1-yl)-4-methylpyridin-2-yl)methoxy)benzyl)-2-(3-iodophenyl)ethan-1-amine* (**17**)

Compound **17** was synthesized by the same method as compound **9**, using 3-iodophenethyl amine as reactant. The product **17** was obtained as pale yellow oil in 87% yield.


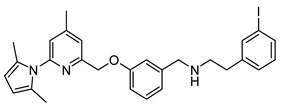


^1^H-NMR (600.15 MHz, DMSO-*d*_6_) δ 7.77 (s, 1H), 7.70 (d, ^3^*J* = 7.8 Hz, 1H), 7.57 (s, 1H), 7.40–7.36 (m, 3H), 7.24 (t, ^3^*J* = 7.8 Hz, 1H), 7.16 (s, 1H), 7.07–7.04 (m, 2H), 5.97 (s, 2H), 5.32 (s, 2H), 3.84 (s, 2H), 2.84 (m, 4H), 2.59 (s, 3H), 2.34 (b, 1H), 2.23 (s, 6H). ^13^C-NMR (150.92 MHz, DMSO-*d*_6_) δ 158.1, 156.4, 150.7, 150.6, 143.5, 142.8, 137.2, 134.5, 130.3, 129.1, 128.1, 127.7, 121.3, 121.1, 120.5, 114.2, 112.8, 106.6, 94.7, 69.7, 52.6, 49.9, 35.2, 20.5, 13.0. HRMS (C_28_H_30_IN_3_O) calcd: 552.1501 [M + H]^+^, found: 552.1504 [M + H]^+^. EA calcd: C (60.98), H (5.48), N (7.62), found: C (61.06 ± 0.02), H (5.56 ± 0.01), N (7.36 ± 0.03).

*6-((3-((3-Fluorophenethylamino)methyl)phenoxy)methyl)-4-methylpyridin-2-amine* (**10**)

This compound was prepared as described in literature [25].


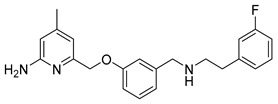


Analytical data correspond to the reported spectroscopic values in literature [25].

*tert-Butyl-(3-((6-(2,5-dimethyl-1H-pyrrol-1-yl)-4-methylpyridin-2-yl)methoxy)benzyl)(3-fluorophenethyl)-carbamate* (**20**)

A stirred mixture of 398 mg (0.90 mmol) of **9**, 218 mg (1.00 mmol) of Boc_2_O and 5 mg (0.04 mmol) of DMAP in 8 mL of methanol was stirred at RT for 15 min. After removal of the solvent under reduced pressure the crude product was purified by flash chromatography (petroleum ether/20% ethyl acetate, *R*_f_ = 0.7) to obtain the product as a colourless oil in 84% yield (410 mg, 0.76 mmol).


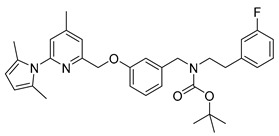


^1^H-NMR (600.15 MHz, DMSO-*d*_6_, mixture of rotamers) δ 7.37 (br, 1H), 7.32–7.28 (m, 1H), 7.26 (t, ^3^*J* = 7.8 Hz, 1H), 7.17 (s, 1H), 7.01–6.95 (m, 3H), 6.94–6.92 (m, 1H), 6.89–6.87 (m, 1H), 6.84–6.83 (m, 1H), 5.78 (s, 2H), 5.15 (s, 2H), 4.34–4.30 (m, 2H), 3.33–3.32 (m, 2H), 2.74–2.73 (m, 2H), 2.39 (s, 3H), 2.03 (s, 6H), 1.32 (s, 9H). ^13^C-NMR (150.92 MHz, DMSO-*d*_6_, mixture of rotamers) δ 162.1 (d, ^1^*J*_CF_ = 243.1 Hz), 158.2, 156.3, 154.8 and 154.5, 150.7, 150.6, 142.1 and 142.1, 140.5 and 140.2, 130.1 (d, ^3^*J*_CF_ = 8.1 Hz), 129.6, 127.6, 124.9 and 124.8, 121.3, 121.0, 120.0 and 119.8, 115.5 and 115.4, 114.0 and 113.6, 113.3, 112.8 (d, ^2^*J*_CF_ = 8.1 Hz), 106.6, 78.8 and 78.7, 69.7, 50.0 and 48.8, 47.6 and 47.5, 33.6 and 33.2, 27.9 and 27.8, 20.5, 13.0. ^19^F-NMR (188.31 MHz, DMSO-*d*_6_, mixture of rotamers) −113.899 and −113.891. HRMS (C_33_H_38_FN_3_O_3_) calcd: 544.2970 [M + H]^+^, found: 544.2970 [M + H]^+^. EA calcd: C (72.40), H (7.04), N (7.73), found: C (72.66 ± 0.08), H (7.18 ± 0.03), N (7.73 ± 0.07).

*tert-Butyl-(3-((6-(2,5-dimethyl-1H-pyrrol-1-yl)-4-methylpyridin-2-yl)methoxy)benzyl)(3-iodophenethyl)carbamate* (**18**)

Compound **18** was synthesized via the same method as compound **20**, using 1.3 g (2.36 mmol) **17** as reactant. The product **18** was obtained as pale yellow oil in 87% yield (1.34 g, 2.05 mmol).


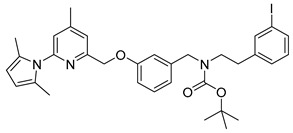


^1^H-NMR (600.15 MHz, DMSO-*d*_6_, mixture of rotamers) δ 7.56–7.53 (m, 2H), 7.37 (s, 1H), 7.26 (t, ^3^*J* = 7.8 Hz, 1H), 7.17–7.13 (m, 2H), 7.07 (t, ^3^*J* = 7.8 Hz, 1H), 6.94–6.92 (m, 1H), 6.90–6.87 (m, 1H), 6.84–6.83 (m, 1H), 5.78 (s, 2H), 5.15 (s, 2H), 4.34–4.29 (m, 2H), 3.31–3.24 (m, 2H), 2.68–2.67 (m, 2H), 2.39 (s, 3H), 2.03 (s, 6H), 1.32 (s, 9H). ^13^C-NMR (150.92 MHz, DMSO*-d*_6_, mixture of rotamers) δ 158.2, 156.3, 154.8 and 154.5, 150.7, 150.6, 142.0 and 141.8, 140.5 and 140.3, 137.4 and 137.3, 134.8, 130.4, 129.6, 128.3 and 128.2, 127.6, 121.3, 121.0, 120.0 and 119.8, 114.0 and 113.6, 113.3, 106.6, 94.9, 78.9 and 78.7, 69.7, 50.0 and 48.7, 47.7 and 47.5, 33.4and 33.1, 27.9 and 27.8, 20.5, 13.0. HRMS (C_33_H_38_IN_3_O_3_) calcd: 652.2031 [M + H]^+^, found: 652.2033 [M + H]^+^. EA calcd: C (60.83), H (5.88), N (6.45). Found: C (60.87 ± 0.11), H (5.90 ± 0.02), N (6.25 ± 0.02).

#### 3.3.3. Synthesis of the Tetrakis(pyridine)copper(II)triflate-complex

This compound was prepared as described in literature [38].


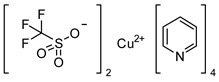


Analytical data correspond to the reported spectroscopic values in literature [38].

#### 3.3.4. Synthesis of the Boronic Ester Precursor *tert*-Butyl(3-((6-(2,5-dimethyl-1*H*-pyrrol-1-yl)-4-methylpyridin-2-yl)methoxy)benzyl)(3-(4,4,5,5-tetramethyl-1,3,2-dioxaborolan-2-yl)phenylethyl)-carbamate (**19**)

A suspension of 420 mg (0.645 mmol) of the iodide **18**, 273 mg (1.074 mmol) of *bis*-pinacolatodiborane, 152 mg (1.548 mmol) of potassium acetate and 15 mg (0.02 mmol) of [1,1′-*bis*(diphenylphosphino)ferrocene]dichloropalladium(II) in 4 mL of DMF was heated at 85 °C for 90 min. The progress of reaction was tested by TLC using 10% ethyl acetate in toluene as mobile phase (*R*_f_ (**18**) = 0.6; *R*_f_ (**19**) = 0.5). After filtration through a Celite^®^ pad and elution with diethyl ether the mixture was concentrated in vacuo at 60 °C. The residue was diluted with diethyl ether, and activated carbon powder and Celite^®^ were added. After stirring for 10 min the suspension was filtered through a Celite^®^/Cellulose (1:1) pad and eluted with diethyl ether. After removal of the solvent under reduced pressure the pale yellow residue was further purified by reversed phase flash chromatography (100% acetonitrile, *R*_f_ = 0.5) to obtain the product as a white solid in 64% yield (271 mg, 0.416 mmol).


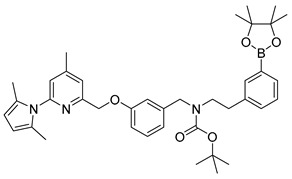


^1^H-NMR (600.15 MHz, *d*_6_-DMSO, mixture of rotamers) δ 7.51–7.49 (m, 2H), 7.36 (br, 1H), 7.29–7.21 (m, 3H), 7.16 (s, 1H), 6.93–6.92 (m, 1H), 6.88–6.86 (m, 1H), 6.83–6.81 (m, 1H), 5.77 (s, 2H), 5.15 (s, 2H), 4.36–4.29 (m, 2H), 3.30 (t, ^3^*J* = 7.2 Hz, 2H), 2.73–2.71 (m, 2H), 2.38 (s, 3H), 2.02 (s, 6H), 1.32 (s, 9H), 1.27 (s, 12H). ^13^C-NMR (150.92 MHz, DMSO-*d*_6_, mixture of rotamers) δ 158.2, 156.3, 154.9 and 154.5, 150.7, 150.6, 140.6 and 140.3, 138.5, 134.9 and 134.8, 132.2, 131.9 and 131.8, 129.6, 128.5, 127.8, 127.6, 121.3, 121.1, 120.0 and 119.7, 113.9 and 113.5, 113.3, 106.6, 83.5, 78.8 and 78.7, 69.7, 50.0 and 48.6, 48.2 and 47.7, 33.8 and 33.5, 27.9 and 27.8, 24.6, 20.5, 13.0. ^11^B-NMR (128.37, *d_6_*-DMSO, mixture of rotamers) δ 28.9 (br). HRMS (C_39_H_50_BN_3_O_5_) calcd: 652.3916 [M + H]^+^, found: 652.3915 [M + H]^+^. EA calcd: C (71.88), H (7.73), N (6.45), found: C (71.53 ± 0.06), H (7.71 ± 0.03), N (6.41 ± 0.03).

## 4. Summary and Conclusions

The synthesis of an ^18^F-labelled aminopyridine derived nNOS-inhibitor was developed here, in order to provide a radioprobe for in vivo imaging studies of the enzyme activity. The preparation of the desired product **[^18^F]10** was successfully achieved by two alternative newer radiosynthesis methods.

The pathway from an iodonium ylide precursor allowed for an efficient nucleophilic ^18^F-functionalisation of an aromatic building block which was further converted to the desired nNOS-inhibitor. Iodonium ylides are ideal precursors for ^18^F-fluorination as they are stable to air and moisture. In order to test for isomerisation processes different positional isomers of the iodonium ylide precursor were synthesized with easy work-up procedures and comparatively high overall yields of about 43%. Their radiofluorination proceeded regioselectively and efficiently with high radiochemical yields of about 78%. The subsequent “build-up” synthesis in 4 steps yielded the desired nNOS-inhibitor **[^18^F]10** in about 15% RCY based on starting [^18^F]fluoride.

Alternatively, with regard to a suitability of the radiofluorination method for automated synthesizers and microfluidic systems, a “late-stage”, two-step procedure for the preparation of the nNOS-tracer was studied based on the copper-mediated ^18^F-fluorination of a corresponding boronic acid ester precursor. The n.c.a. ^18^F-exchange of a dioxaborolane group succeeded with radiochemical yields of up to 53%. A microwave assisted displacement of protecting groups then led to about 15% RCY of **[^18^F]10**, based on starting [^18^F]fluoride. Thus, the present study is also a further example for the applicability of this relatively new, very promising n.c.a. radiofluorination method.

So far, there exist only a few effective “late-stage” labelling methods for n.c.a. ^18^F-fluorination of non-activated arenes. Often, those are limited by the preparative possibilities of suitable, corresponding precursors, especially of highly functionalized compounds. Considering that this study focused on the preparation of the inhibitor **[^18^F]10** for further preclinical evaluation, both labelling routes were not optimized to the same level, and thus a conclusive comparison of the methods cannot be given. This would demand an evaluation of many more compounds ^18^F-labelled by those processes under comparable conditions and efforts, respectively. Following remarks, however, might be of interest.

Beneficially, ylide precursors are easy to handle and render high radiofluorination yields which are possible without the need of metal catalysts. However, their production requires oxidative conditions, why nitrogen containing compounds sometimes cannot be converted into their corresponding iodine(III)-intermediates. A recent publication on radiofluorination of spirocyclic iodonium ylides, however, demonstrated the possibility to produce precursors from a variety of nitrogen- and oxygen-containing heterocycles [39], expanding the scope of electron-rich and sterically hindered [^18^F]fluoroarenes [40]. Further on, ylides of complex precursors were already shown for some time to be accessible [41]. Thus, both types of precursors used here would in principle allow the nucleophilic ^18^F-exchange in a late synthetic step. Indeed, the iodinated intermediate **18** supposedly enables its conversion to a spirocyclic iodonium ylide as well as to a dioxaborolane. This would allow to directly compare both types of precursors in further studies on a “late-stage” radiofluorination of compound **10**.

The chemoselective preparation of a corresponding dioxaborolane as precursor offers probably complimentary possibilities to ylides, depending on the composition of functional groups of the compound to be labelled. At the end, the composition of the compound will determine the accessibility of a desired ylide or boronic acid precursor. In this study it was found that the borylation conditions should be carefully optimized and that highly reactive iodides lend preferably to reach high yields and pure products. In contrast, when starting from corresponding bromides, the isolation of the product was difficult after the Miyaura borylation, if the conversion was not quantitatively completed.

At the present stage of optimization devoted, the radiochemical yield of the nNOS inhibitor **[^18^F]10**, after “build-up” process and “late-stage” labelling, was directly comparable. Although potential for improvement seems warranted, both methods sufficiently provide the desired inhibitor as probe for further preclinical imaging studies with PET, aiming at the determination of the activity of nNOS.

## Figures and Tables

**Table 1:** Employed conditions of high performance liquid chromatography (HPLC) separations.

HPLC Condition	Column	Eluent	Wavelength	Flow	Injection-Volume
**1**	c	40 MeCN:60 H_2_O	254 nm	1 mL/min	20 µL
**2**	b	60 MeCN:40 H_2_O	261 nm	1 mL/min	20 µL
**3**	c	^a^ A: H_2_O + 0.1% TEA, B: MeCN + 0.1% TEA	261 nm	1 mL/min	20 µL
**4**	a	70 MeCN:30 H_2_O + 0.1% TEA	261 nm	1 mL/min	20 µL
**5**	a	60 ACN:40 H_2_O + 0.1% TEA	261 nm	1 mL/min	20 µL
**6**	a	**^b^** A: H_2_O + 0.1% TEA B: MeCN + 0.1% TEA	261 nm	1 mL/min	20 µL
**7**	a	^c^ A: H_2_O + 0.1% TEA B: MeCN + 0.1% TEA	261 nm	1 mL/min	20 µL

^a^ gradient program of method 3: 0–16 min 70% A, 16–30 min 70%–50% A, 30–40 min 50% A, 40–41 min 50%–70% A, 41–60 min 70% A; ^b^ gradient program of method 6: 0–10 min 40% A, 10–15 min 40%–20% A, 15–35 min 20% A, 35–40 min 20%–40% A, 40–60 min 40% A; ^c^ gradient program of method 7: 0–8 min 40% A, 8–15 min 40%–20% A, 15–40 min 20% A, 40–45 min 20%–40% A, 45–60 min 40% A.

**Table 2:** *k*-Values of ^19^F-reference compounds for HPLC-identification of labelled compounds.

Compound	HPLC-System	Condition	*k*-Values
Fluoroanisole	Knauer	1	6.7 (anisole), 7.3 (2-fluoroanisole), 9.3 (3-fluoroanisole), 8.2 (4-fluoroanisole)
**15**	Knauer	2	3.2
**21o**	Dionex	3	6.0
**21m**	Dionex	3	7.0
**21p**	Dionex	3	8.0
**9**	Knauer	4	6.7
**9**	Knauer	5	19.4
**10**	Knauer	5	2.9
**9**	Dionex	6	7.3
**10**	Dionex	6	2.7
**9**	Dionex	7	2.7
**10**	Dionex	7	7.2
**20**	Dionex	7	14.0

**Table 3:** *R*_f_-values of relevant reference compounds for radio-thin layer chromatography (TLC) analyses.

Compound	Eluent	*R*_f_
**9**	9 EE:1 PE + 1% TEA	0.8
**10**	9 EE:1 PE + 1% TEA	0.5
**15**	1 EE:4 PE	0.5
**20**	1 EE:4 PE	0.7
